# Constitutively active PIK3CA mutations are expressed by lymphatic and vascular endothelial cells in capillary lymphatic venous malformation

**DOI:** 10.1007/s10456-020-09722-0

**Published:** 2020-04-30

**Authors:** Timothy D. Le Cras, Jillian Goines, Nora Lakes, Patricia Pastura, Adrienne M. Hammill, Denise M. Adams, Elisa Boscolo

**Affiliations:** 1 Division of Pulmonary Biology, Cincinnati Children’s Hospital Medical Center, 3333 Burnet Avenue, Cincinnati, OH 45229-3039 USA; 2 Department of Pediatrics, University of Cincinnati College of Medicine, Cincinnati, OH USA; 3 Division of Experimental Hematology and Cancer Biology, Cincinnati Children’s Hospital, Cincinnati, OH USA; 4 Cancer and Blood Diseases Institute, Division of Hematology, Cincinnati Children’s Hospital, Cincinnati, OH USA; 5 Boston Children’s Hospital Division of Hematology/Oncology Harvard Medical School, Boston, MA USA

**Keywords:** Vascular anomaly, Lymphatic malformation, PI3K, AKT, Endothelial cell, Vascular, Patient-derived xenograft

## Abstract

Capillary lymphatic venous malformations (CLVM) are complex vascular anomalies characterized by aberrant and enlarged lymphatic and blood vessels. CLVM appear during fetal development and enlarge after birth, causing life-long complications such as coagulopathy, pulmonary embolism, chronic pain, and disfigurement. Treatment includes surgical debulking, amputation, and recurrent sclerotherapy. Somatic, mosaic mutations in the 110-kD catalytic α-subunit of phosphoinositide-3-kinase (PIK3CA) gene have been previously identified in affected tissues from CLVM patients; however, the cell population harboring the mutation is still unknown. In this study, we hypothesized that endothelial cells (EC) carry the *PIK3CA* mutations and play a major role in the cellular origin of CLVM. We isolated EC from the lesions of seven patients with CLVM and identified *PIK3CA* hotspot mutations. The CLVM EC exhibited constitutive phosphorylation of the PI3K effector AKT as well as hyperproliferation and increased resistance to cell death compared to normal EC. Inhibitors of PIK3CA (BYL719) and AKT (ARQ092) attenuated the proliferation of CLVM EC in a dose-dependent manner. A xenograft model of CLVM was developed by injecting patient-derived EC into the flanks of immunocompromised mice. CLVM EC formed lesions with enlarged lymphatic and vascular channels, recapitulating the patient histology. EC subpopulations were further obtained by both immunomagnetic separation into lymphatic EC (LEC) and vascular EC (VEC) and generation of clonal populations. By sequencing these subpopulations, we determined that both LEC and VEC from the same patient express the *PIK3CA* mutation, exhibit increased AKT activation and can form lymphatic or vascular lesions in mouse.

**Electronic supplementary material:**

The online version of this article (10.1007/s10456-020-09722-0) contains supplementary material, which is available to authorized users.

## Introduction

The vascular and lymphatic systems are highly interconnected and arise from common precursor cells. During mouse development, lymphatic vessels can originate from a subset of endothelial cells expressing PROX1 (Prospero Homeobox-1) in the cardinal vein [1–3]. Genetic defects that arise during the development of the vascular and/or lymphatic systems are associated with a variety of clinical vascular anomalies [4].

Capillary lymphatic venous malformations (CLVM) [5, 6] are characterized by malformed hyperplastic capillaries, lymphatics and veins. CLVM appear during embryonic or fetal development, enlarge with time and never regress. The proportion of different vessel types in CLVM—capillaries, lymphatics and veins—varies greatly between patients [7, 8]. The abnormal vasculature in CLVM can cause significant and long-term morbidity including a multitude of complications, such as recurrent infections, chronic pain, thrombosis, and/or bleeding. Disease management in these patients is life-long and consists of recurrent sclerotherapy procedures, surgical debulking and even amputation [9]. These standard-of-care therapies are highly invasive and only manage symptoms rather than targeting the disease etiology. Recently, pharmacologic treatment with the mTOR inhibitor Sirolimus (rapamycin) has shown some efficacy in treating CLVM [10–12]. However, rapamycin is an immunosuppressant and the toxicity associated with long-term treatment of children is of particular concern. Hence, deciphering the cellular and molecular pathways driving CLVM pathogenesis is paramount to identify targeted therapies that lead to reversal and long-term arrest of the disease process.

CLVM can occur in individuals with overgrowth syndromes such as Klippel-Trenaunay Syndrome (KTS) [13, 14] and Congenital Lipomatous Overgrowth, Vascular malformations, Epidermal nevi, Scoliosis/skeletal and spinal syndrome (CLOVES) [15, 16]. Recent studies have identified somatic, mosaic mutations in the 110-kD catalytic α-subunit of phosphoinositide-3-kinase (*PIK3CA*) in these patients’ lesions [17–19]. Mutations in these same hotspot regions of *PIK3CA* are also found in cancer, developmental tissue overgrowth syndromes termed *PIK3CA*-Related Overgrowth Spectrum (PROS), and simple lymphatic and venous malformations [20–26]. These *PIK3CA* mutations are gain-of-function and drive constitutive PI3K/AKT (Protein Kinase B, PKB)/mTOR (mammalian Target of Rapamycin) signaling, thereby increasing cell survival, proliferation and angiogenesis [21, 24, 27–30]. While *PIK3CA* mutations have been identified in lesion tissue from individuals with CLVM, including KTS and CLOVES [17, 18, 23], their exact cellular location is still poorly defined. Recent studies of LM and VM show that the genetic variant is specific to the EC [20, 25, 27, 29, 31].

In this study, we hypothesized that *PIK3CA* mutations originate in the EC population of CLVM lesions. To test our hypothesis, we devised a protocol to isolate and purify EC from both resected human CLVM lesion tissue and sclerotherapy fluid. After confirming pure separation of EC, we investigated the presence of *PIK3CA* mutations in the EC and genomic DNA from 7 CLVM patients. Furthermore, we assessed the signaling pathways downstream of the mutant *PIK3CA* and the proliferative potential of CLVM EC. To study the in vivo contribution to disease, we generated a xenograft model by injecting patient-derived EC into mice. In this model, we evaluated the capacity of CLVM EC to form abnormal vascular and lymphatic channels. Patient-derived EC were further separated into lymphatic EC (LEC) and non-lymphatic, vascular EC (VEC) and DNA sequenced to determine if *PIK3CA* mutations are present in both EC populations. We further assessed the ability of mutant single cell-derived VEC and LEC clonal populations to generate vascular and lymphatic lesions in the xenograft model.

## Materials and methods

The authors declare that all supporting data are available within the article.

### Tissue samples

Patient tissue samples were obtained from participants after informed consent from the Collection and Repository of Tissue Samples and Data from Patients with Tumors and Vascular Anomalies (IRB #2008-2001 and IRB #2016-3878 per institutional policies) at Cincinnati Children’s Hospital Medical Center, Cancer and Blood Disease Institute and with approval of the Committee on Clinical Investigation. Collected data and identifying names were stored in a secure database maintained by the Cancer and Blood Disease Institute. This was further deidentified by creating a patient ID for use in this study. Table 1 lists relevant patient sample information (*n* = 7). Samples included excised tissue, blood/fluid aspirated during sclerotherapy, and peripheral blood.

**Table 1:** Patient information

ID	Diagnosis/clinical phenotype	Affected area	Age at collection	Source of cells
A	CLVM/KTS	Pelvis, perineum, right leg	4 years	Tissue
B	CLVM/NOS*	Left leg (subcutaneous, intramuscular, intraosseous)	11 years	Tissue
C	CLVM/CLOVES	Trunk, bilateral legs	4 years	Tissue
D	CLVM/CLOVES	Trunk, right arm	8 months	Lesion blood
E	CLVM/CLOVES	Brain, face, trunk, extremities	2 years	Tissue
F	CLVM/KTS	Right leg	21 years	Lesion blood
G	CLVM/CLOVES	Left arm, chest/abdomen/pelvis	1 year	Tissue

^*^Not otherwise specified—without overgrowth, does not fit PROS, KTS, or CLOVES

### Cell culture and isolation

Patient-derived cells were isolated from solid tissue and lesion fluid as previously described [29]. In brief, tissue was homogenized, and the cell pellet was collected by centrifugation, resuspended in Endothelium Growth Medium-2 (EGM-2) (Lonza) supplemented with 20% fetal bovine serum (FBS) (Hyclone) and 1% penicillin–streptomycin-glutamine (PSG) (Corning), and seeded onto fibronectin-coated (Millipore) plates. When cells reached 80% confluency, endothelial cells (EC) were isolated with anti-CD31-conjugated magnetic beads (Dynal) according to manufacturer’s instructions. After confirming pure CD31^+^ expression by immunofluorescence, EC were further separated into vascular EC (VEC) (CD31^+^ /D2-40^−^) and lymphatic EC (LEC) (CD31^+^/D2-40^+^) populations. CD31^+^ cells were incubated with anti-D2-40 primary antibody (1:40 Biolegend) for 45 min, followed by selection using anti-mouse IgG-conjugated magnetic beads (Dynal) according to manufacturer’s instructions. VEC and LEC clones were obtained by diluting and seeding CLVM D EC at early passage (p2) with one cell on average for every 5 wells in 96-well plates. Human dermal lymphatic endothelial cells (HDLEC), human umbilical vein endothelial cells (HUVEC), foreskin EC, and human lung fibroblasts served as healthy controls and were obtained from Lonza. All cell types were cultured in identical conditions unless otherwise noted: EGM-2 supplemented with 20% FBS and 1% PSG, on fibronectin-coated plates at 37 °C, 5% CO_2_. Cells were used between passages 1 and 10.

### DNA sequencing

CLVM cell populations were assessed for hotspot mutations in the PIK3CA gene via Sanger Sequencing. DNA was extracted from pure CLVM EC, VEC and LEC populations using a QIAamp DNA Mini Kit (Qiagen). Genomic DNA was extracted from peripheral blood using a Pure-link Genomic DNA Mini Kit (Invitrogen). DNA quality and quantity were assessed using a Nanodrop 2000c Spectrophotometer. Primers were used to amplify *PIK3CA* exons 7, 9, and 20 (Integrated DNA Technologies). Amplified product was purified using QIAquick Gel Extraction Kit (Qiagen) and sequenced by the CCHMC DNA Sequencing and Genotyping Core. Electropherogram results were visualized using CodonCode Aligner (CodonCode Corporation).

### Immunofluorescence staining

Cell monolayer phase photographs were acquired using a phase-contrast microscope (Zeiss) with ZenLite software. Immunofluorescence staining was performed on monolayers once the cells reached 80% confluency. To visualize PROX1 and VE-Cadherin, monolayers were fixed in 4% paraformaldehyde (Electron Microscopy Sciences) at room temperature for 15 min. Cells were pre-blocked and permeabilized with 1% bovine serum albumin (BSA) (Sigma)/ 0.3% Triton X-100 (Sigma) in Phosphate Buffered Saline (PBS) (Fisher) for 1 h. Primary incubation with anti-VE-Cadherin (1:50, Santa Cruz) or anti-PROX1(1:50, R&D Systems) was performed for 1 h. To visualize CD31, CD90, and D2-40, monolayers were fixed in methanol (Fisher) at 4 °C for 10 min. Primary antibody incubation with anti-CD31 (1:50, Dako), anti-CD90 (1:100, BD Biosciences), or anti-D2-40 (1:50, Biolegend) was performed for 1 h. Secondary antibody incubation with fluorescein isothiocyanate (FITC) or Texas Red-conjugated secondary antibodies (1:200, Vector Laboratories) was done for 1 h. Samples were mounted using Prolong Gold with 4′,6-diamidino-2-phenylindole (DAPI) (Life Technologies) and imaged with a C2 confocal microscope (Nikon).

Sections of paraffin-embedded tissue from human CLVM, hemangioma (vascular tumor), lymphatic malformation, healthy foreskin, mouse skin, and xenograft lesions were stained with hematoxylin and eosin (H&E). Sections underwent antigen retrieval using 0.01 mM Tris/ 0.5 M EDTA buffer (pH 9.0) and were blocked using 5% horse serum (Vector Laboratories) in PBS. Frozen tissue from human CLVM, human hemangioma, and healthy foreskin were blocked using 1% BSA/ 0.3% Triton X-100/ 5% goat serum (Vector Laboratories). Immunofluorescence was performed using biotin-conjugated Ulex europaeus agglutinin I (UEA) (1:100, Vector Laboratories), human-specific anti-D2-40 (1:50, Biolegend), mouse-specific anti-D2-40 (1:100, Santa Cruz), biotin-conjugated Isolectin B4 (IB4) (1:100, Vector Laboratories), phospho-Ser473-AKT (1:200, Cell Signaling), and anti-CD31 (1:50, Dako) primary antibodies, followed by Texas Red- and FITC-conjugated secondary antibodies (1:200, Vector Laboratories). Nuclei were stained with DAPI (1:1000, Life Technologies). Samples were mounted using Prolong Gold Antifade (Life Technologies) and imaged with a C2 confocal microscope.

### Immunoblotting

CLVM EC, HUVEC, and HDLEC were expanded in EGM-2 supplemented with 20% FBS. Cells were washed with PBS and lysed using RIPA buffer (Teknova) with phosphatase and protease inhibitor cocktail (Roche). Cell lysates were separated by electrophoresis and transferred to polyvinylidene difluoride membranes (Invitrogen). Membranes were probed with the following primary antibodies: VEGFR-2 (1:1000, Cell Signaling Technology), PROX1 (1:200, R&D Systems), VEGFR-3 (1:250, Millipore), phospho-Ser473-AKT (1:2000), phospho-Thr308-AKT (1:1000), AKT (1:1000), phospho-ERK 1/2 (1:2000), ERK 1/2 (1:1000) (all these antibodies from: Cell Signaling Technology), and β-Actin (1:10000, Sigma). Membranes were incubated with peroxidase-conjugated secondary antibodies (1:10000, Calbiochem). Antigen-antibody complexes were visualized using Immobilon Forte Western HRP Substrate (Millipore) and ImageQuant LAS 4000 (GE Healthcare). Band intensity was quantified using ImageJ software.

### Xenograft model for CLVM

All animal experiments were conducted according to a protocol approved by the Institutional Animal Care and Use Committee at Cincinnati Children’s Hospital in an Association for Assessment and Accreditation of Laboratory Animal Care-approved facility. All methods for mice were performed in accordance with the guidelines and regulations in the approved protocol. Following expansion in tissue culture, 2.5–3.0 × 10^6^ CLVM EC (or VEC and LEC clones), HUVEC, and HDLEC were suspended in 200μL Matrigel® (Corning) and subcutaneously injected into both flanks of 6 to 7-week-old male athymic nu/nu male mice (Envigo). Lesions were dissected 9–11 days later, fixed in 10% formalin, and paraffin embedded. Sections (5 μm) were stained with hematoxylin and eosin (H&E). Five random images per section were captured using an EVOS microscope (Life Technologies), and vascular area (%) was quantified using ImageJ software.

### Cell proliferation

Cell proliferation was measured using the SRB (Sulforhodamine B) assay [32]. In brief, cells were seeded at 6000 cells per well in fibronectin-coated 96-well plates and cultured in EGM-2 supplemented with 20% FBS. After 12 h (time point = 0 h), wells were washed with PBS and replaced with either EGM-2 supplemented with 20% FBS, or Endothelial Basal Medium (EBM-2) (Lonza) without FBS. At each time point, plates were washed with PBS and fixed with 10% trichloroacetic acid (Sigma). Cells were washed with diH_2_O, dried, and incubated with 0.4% SRB (Sigma) in 0.1% acetic acid (Fisher) for 15 min. After washing with 0.1% acetic acid and drying, SRB was solubilized in 150μL of 10 mM Tris base (pH 10.5) (Biorad) per well, and the absorbance at 554 nm was read using a FlexStation3 Microplate Reader (Molecular Devices). Proliferative capacity was calculated by [fold change = (mean absorbance at time point) / (mean absorbance at 0 h)].

### Cell death

Resistance of CLVM EC to cell death stimuli was measured using an IncuCyte Live Cell Analysis system (Essen Bioscience). Cells were seeded at 6000 cells per well in fibronectin-coated 96-well plates and cultured in EGM-2 supplemented with 10% FBS. After 12 h (time point = 0 h), wells were washed with PBS and replaced with 250 nM Cytotox Red reagent (Essen Bioscience) in EBM-2 without FBS. Images were acquired every 4 h, and cell death was calculated as follows: $\left[ {{\text{fold change}} = \left( {{\text{number Cytotox}}^{ + } {\text{cells at time point}}} \right)/\left( {{\text{number Cytotox}}^{ + } {\text{cells at }}0{\text{ hours}}} \right)} \right]$ .

### Drug treatment

Cells were seeded at 6000 cells per well in fibronectin-coated 96-well plates and cultured in EGM-2 supplemented with 20% FBS. After 12 h (time point = 0 h), the media was replaced with EBM-2 supplemented with 10% FBS and the indicated drugs: BYL719, ARQ092, U0126 (1,5,10 μM), or rapamycin (15 nM). DMSO was the vehicle-only control. Cell proliferation was measured at 48 h using the SRB assay. The percent (%) inhibitory rate of compounds was calculated as $\left[ {\left( {\left( {{\text{OD}}_{{{54}0}} {\text{untreated}}{-\!\!-}{\text{OD}}_{{{54}0}} {\text{compound}}} \right)/\left( {{\text{OD}}_{{{54}0}} {\text{untreated}}} \right)} \right)*{1}00\% } \right]$ .

### Flow cytometry

Following expansion in tissue culture, 1.0 × 10^5^ CLVM EC, HUVEC, and HDLEC were detached and resuspended in cold PBS supplemented with 0.5% BSA and 2 mM EDTA. Cells were incubated with CD31-FITC (1:100, BD Pharmingen), VEGFR-3-PE (1:100, R&D Systems), or CD45-PE (1:200, BD Biosciences) at 4 °C for 20 min, washed 3 times, and stored in PBS supplemented with 1% paraformaldehyde. Flow cytometry was performed using BD FACSCanto or LSR Fortessa and analyzed with BD FACSDiva 8.0.1 or FlowJo software.

### Statistical analysis

Cell proliferation was analyzed using a repeated measures two-way ANOVA with Greenhouse-Geisser correction, followed by Holm Sidak’s multiple comparison test. Cell death was analyzed using a two-way ANOVA followed by Holm Sidak’s multiple comparison test. Drug studies were analyzed using a one-way ANOVA, followed by Holm Sidak’s multiple comparison test. In vivo vascular area and western blot band quantifications were analyzed using Welch’s *t*-test. All calculations were performed using GraphPad Prism. Differences were considered significant if *p*-value $\le$ 0.05.

## Results

### Isolation and characterization of EC from CLVM lesions

**Fig. 1:**
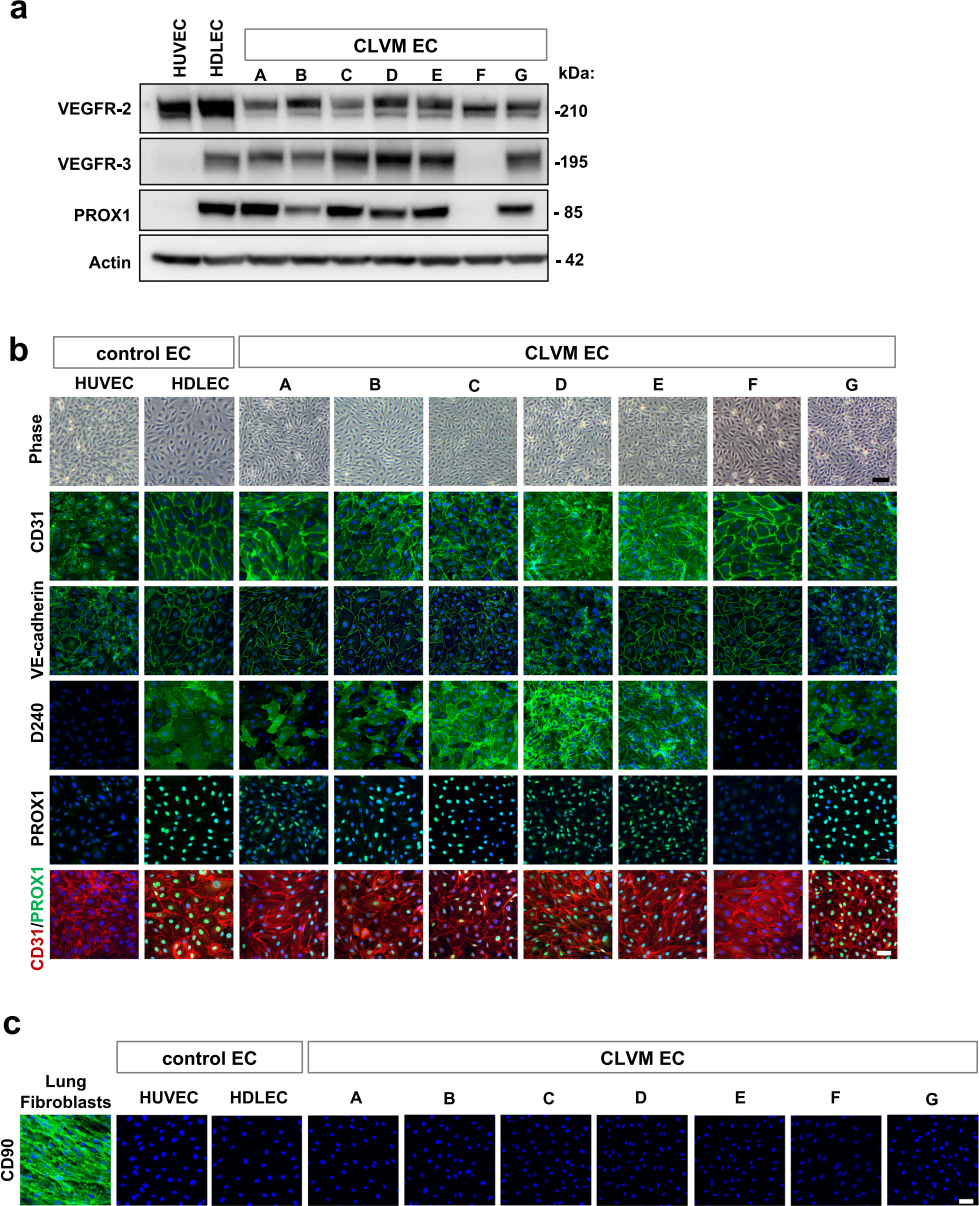
Characterization of patient-derived CLVM EC. **a** Western blot analysis for pan-EC marker VEGFR-2 and LEC-specific markers VEGFR-3 and PROX1 in patient-derived CLVM EC, human umbilical vein endothelial cells (HUVEC), and human dermal lymphatic endothelial cells (HDLEC). β-Actin serves as loading control. **b** Contrast phase and immunofluorescence images of CLVM EC at 80–90% confluency stained for pan-EC markers CD31 and VE-Cadherin, and LEC-specific markers D2-40 and PROX1. EC and LEC markers (green/red), nuclei (DAPI, blue). Scale bar: phase 100 µm; immunofluorescence 50 µm. **c** Immunofluorescence images of lung fibroblasts, HUVEC, HDLEC and CLVM EC at 80–90% confluency stained for the stem cell/fibroblast marker CD90 (green) and DAPI for nuclei (blue). Scale bar: 50 µm

EC were isolated from the lesions of 7 CLVM patients, with 5 samples derived from solid tissue and 2 from fluid collected during sclerotherapy (Table 1). Most CLVM EC expressed both vascular (VEGFR-2) and lymphatic (VEGFR-3, PROX1) markers (Fig. 1a). CLVM EC exhibited a typical endothelial, cobblestone morphology as well as expression of the EC-specific markers CD31 and VE-Cadherin and lymphatic markers D2-40 and PROX1 (Fig. 1b). Furthermore, co-staining of PROX1 (or VEGFR-3) and CD31 showed that CLVM EC populations are composed of both LEC (PROX1+/CD31+), and VEC (PROX1-/CD31+), although at variable ratios (Fig. 1b, and Suppl. Fig. S1a for VEGFR-3/CD31 colocalization by flow cytometry). In addition, we demonstrate that our CLVM EC populations did not express the fibroblast/stem cell marker CD90 (Fig. 1c) nor the hematopoietic cell marker CD45 (Suppl. Fig. S1b). These data indicate that CLVM EC are comprised of both LEC and VEC in varying proportions.

### Somatic hotspot PIK3CA mutations are present in CLVM EC

**Fig. 2:**
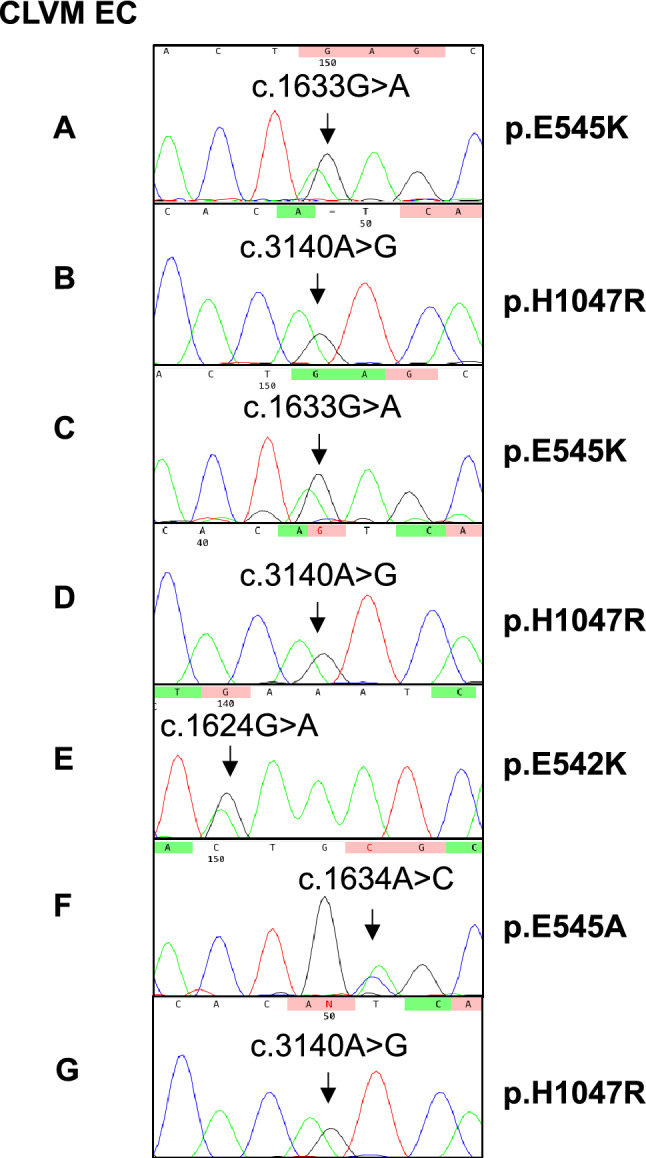
DNA Sanger sequencing of CLVM EC revealed hotspot mutations in *PIK3CA*. **a** Sequencing for each CLVM EC showing chromatograms for c.1633G > A (p.E545K), c.3140A > G (p.H1047R), c.1624G > A (p.E542K), and c.1634A > C (p.E545A)

DNA was extracted from CLVM EC populations and genomic DNA from peripheral blood. Sanger sequencing was performed on *PIK3CA* exons 7 (C2 domain), 9 (alpha helical domain), and 20 (kinase domain) to detect activating mutations (specifically at p.C420, E542, E545, and H1047). Heterozygous *PIK3CA* hotspot mutations were identified in all 7 CLVM EC (Fig. 2) and, as expected, no *PIK3CA* variants were found in the genomic DNA. These data confirm that somatic *PIK3CA* mutations are present in lesion CLVM EC, suggesting that these cells are key drivers of the vascular anomaly.

### CLVM EC display constitutive AKT activation and hyperproliferation

**Fig. 3:**
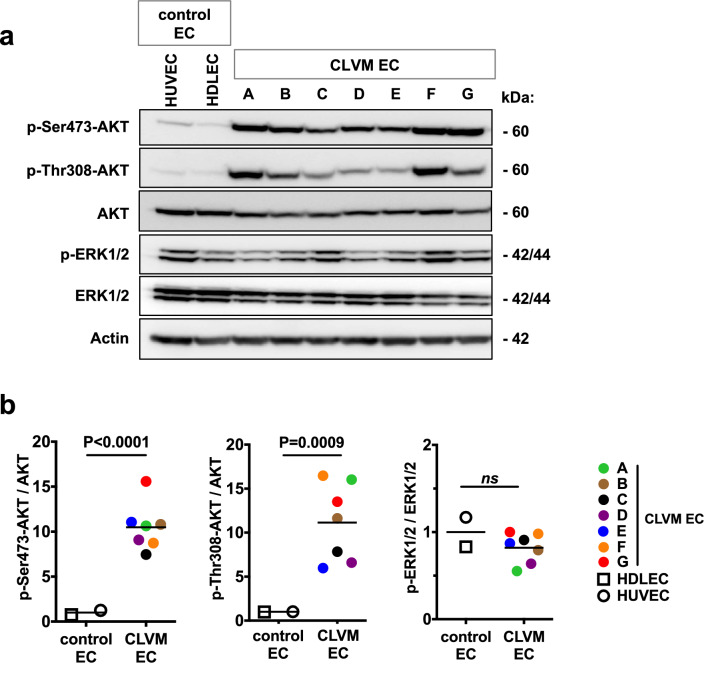
Increased PI3K-AKT signaling in CLVM EC. **a** Western blot analysis of AKT (Ser473), AKT (Thr308), and ERK1/2 phosphorylation in CLVM EC compared to normal HUVEC and HDLEC. β-Actin serves as loading control. **b** Densitometric quantification of phospho-AKT(Ser473), phospho-AKT(Thr308), phospho-ERK1/2 western blot bands relative to total AKT and ERK1/2. Horizontal bar shows mean, data is normalized to control EC mean, Welch’s *t*-test

**Fig. 4:**
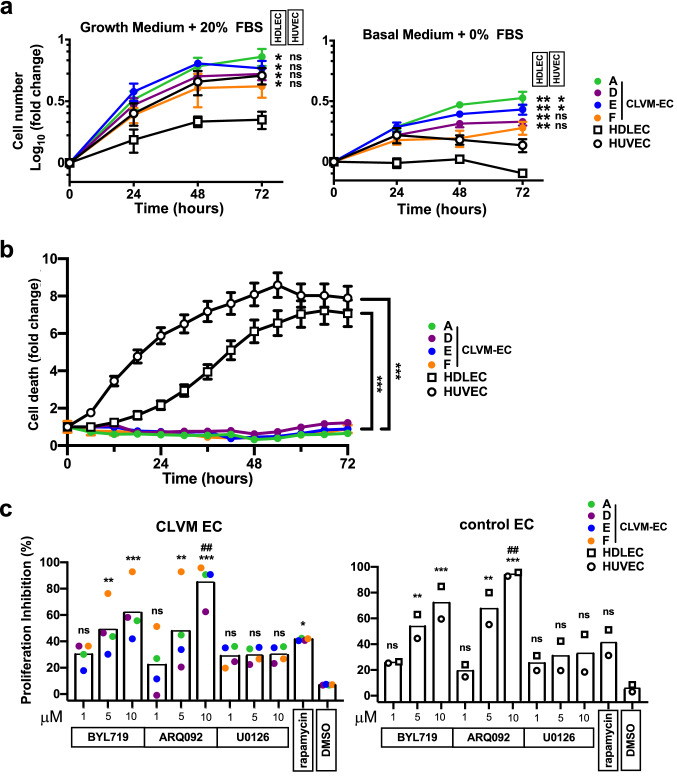
Proliferative capacity of CLVM EC and response to PI3K-AKT inhibition. **a** Proliferation rates of CLVM EC, HDLEC and HUVEC in growth medium with 20% fetal bovine serum (FBS) (left) and in basal medium without FBS (right); (*n* = 3–4 independent experiments), two-way ANOVA. *P < 0.05, **P < 0.01 **b** Resistance of CLVM EC and control EC to cell death induced by growth factor withdrawal (representative experiment, *n* = 3), two-way ANOVA. ***P < 0.001. The cell death rate was measured as [(number of cytotox+cells at time analyzed)/ (number of cytotox+cells at time 0)]. **c** CLVM EC (CLVM A, D, E, F) and control EC (HDLEC and HUVEC) treated for 48hs with PIK3CA inhibitor BYL719, AKT inhibitor ARQ092, MEK inhibitor U0126 (1, 5, 10 μM) and rapamycin (15 nM). Horizontal bar shows mean, data is normalized to untreated control. The percent (%) inhibitory rate of compounds was calculated as [((OD_540_ untreated—OD_540_ compound) / (OD_540_ untreated)) * 100%]. One-way ANOVA, **P < 0.01, ***P < 0.001 versus DMSO, ^##^*P* < 0.01 versus rapamycin, ns for *P* > 0.05 versus DMSO or rapamycin

Using protein immunoblotting, phospho-AKT levels were measured in CLVM EC lysates to assess signaling targets downstream of PI3K. CLVM EC displayed a 11-fold increase in AKT phosphorylation at both the Ser473 and Thr308 residues (Fig. 3a, b), compared to normal EC (HUVEC and HDLEC). Furthermore, there was no difference in ERK1/2 phosphorylation levels between mutant CLVM EC and normal EC. Next, the proliferative capacity of CLVM EC (we selected one CLVM EC for each *PIK3CA* mutation type) was assessed over 72 h. CLVM EC cultured in presence of endothelial-specific growth factors displayed increased proliferation (*p* < 0.05) compared to HDLEC but not HUVEC (Fig. 4a). Notably, CLVM EC cultured in the absence of growth factors and serum continued to proliferate over time (*p* < 0.01), while after the 24 h time point, HDLEC and HUVEC numbers remained static and ultimately decreased (Fig. 4a). To further investigate the hyperproliferative nature of CLVM EC, we exposed the cells to cell death-inducing stimulus (basal medium without growth factors and serum) and found that CLVM EC are largely resistant to cell death, while normal EC display an eightfold increase in cell death over 72 h (*p* < 0.001) (Fig. 4b and Suppl. Fig. 2a, b). Although proliferation was assessed in CLVM EC with different *PIK3CA* mutations, we did not detect significant differences in the proliferation rates or AKT activation that could be attributed to the hotspot location. Together, these findings suggest that hotspot *PIK3CA* mutations in CLVM EC cause constitutive activation of the PI3K-AKT pathway, thereby promoting hyperproliferation and resistance to cell death, even in the absence of growth factors.

To further validate the role of hyperactive PI3K/AKT signaling in CLVM EC, we tested the effect of PIK3CA inhibitor BYL719 and AKT inhibitor ARQ092. Both BYL719 and ARQ092 showed a dose-dependent inhibition of cell proliferation in CLVM EC, while the MEK inhibitor U0126 did not have a significant effect (Fig. 4c and Suppl. Fig. S3a) compared to vehicle treated cells. Of note, the AKT inhibitor ARQ092 at 10 μM showed significantly (*P* < 0.01) increased potency in inhibiting cell proliferation when compared to rapamycin and resulted in increased cell death in CLVM EC compared to control EC (Suppl. Fig. S3b).

### Patient-derived CLVM EC form enlarged vascular and lymphatic channels in a xenograft model

**Fig. 5:**
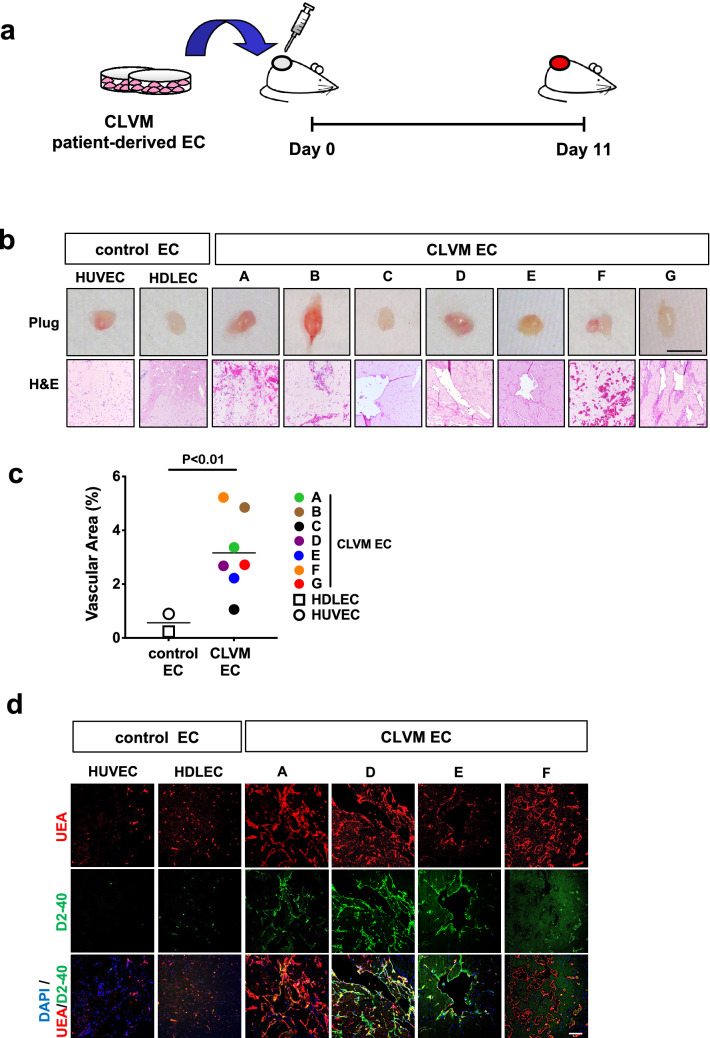
Characterization of CLVM patient-derived EC xenograft model. **a** Schematic: patient-derived CLVM EC were suspended in Matrigel® and injected subcutaneously into the flank of immunocompromised mice. **b** On day 11, lesion plugs from control EC (HUVEC and HDLEC) and CLVM EC were sectioned and stained with hematoxylin and eosin (H&E). Scale bars: plug 1 cm; H&E 100 µm; **c** Vascular area quantification as % of channel area over total area. (5 fields/lesion. *n* = 7–8 mice with 2 lesions each, per group; 7/7 CLVM EC were analyzed, Welch’s *t*-test). Horizontal bars show mean. **d** Sections from the center of Matrigel plugs were stained with human-specific EC lectin Ulex europaeus agglutinin I (UEA) (red), and LEC marker D2-40 (human-specific antibody) (green). Scale bar: 100 μm

CLVM EC from the 7 patients were expanded in culture, suspended in Matrigel and subcutaneously injected into immune-deficient mice to evaluate if they could recapitulate the histological features of CLVM tissue (Fig. 5a). Murine lesion xenograft explants (plugs) were visibly vascularized on day 11 (Fig. 5b). Hematoxylin and eosin and immuno- staining of plug sections revealed numerous enlarged vascular and lymphatic channels, while injection of HUVEC and HDLEC into mouse generated only minimal vascularization compared to CLVM EC (Fig. 5b). The vascular area in CLVM EC xenograft lesions was significantly higher than control EC (*p* < 0.01) (Fig. 5c). The CLVM EC xenograft lesions recapitulated the patient histology (shown for reference in Suppl. Fig. S4). Lesional ectatic channels in murine CLVM showed expression of both D2-40 and the human pan-endothelial agglutinin Ulex europaeus agglutinin I (UEA) or UEA only (Fig. 5d), suggesting that both LEC (UEA^+^ /D2-40^+^) and VEC (UEA^+^ /D2-40^−^) contribute to the dysmorphic vasculature in proportions that vary between patients. Furthermore, we confirmed that channels were generated by the human CLVM EC rather than invading mouse vessels by utilizing human-specific lectin and antibody (UEA and D2-40) (see Suppl. Fig. S5 for staining controls).

### Somatic hotspot *PIK3CA* mutations are present in both lymphatic and vascular EC in CLVM

**Fig. 6:**
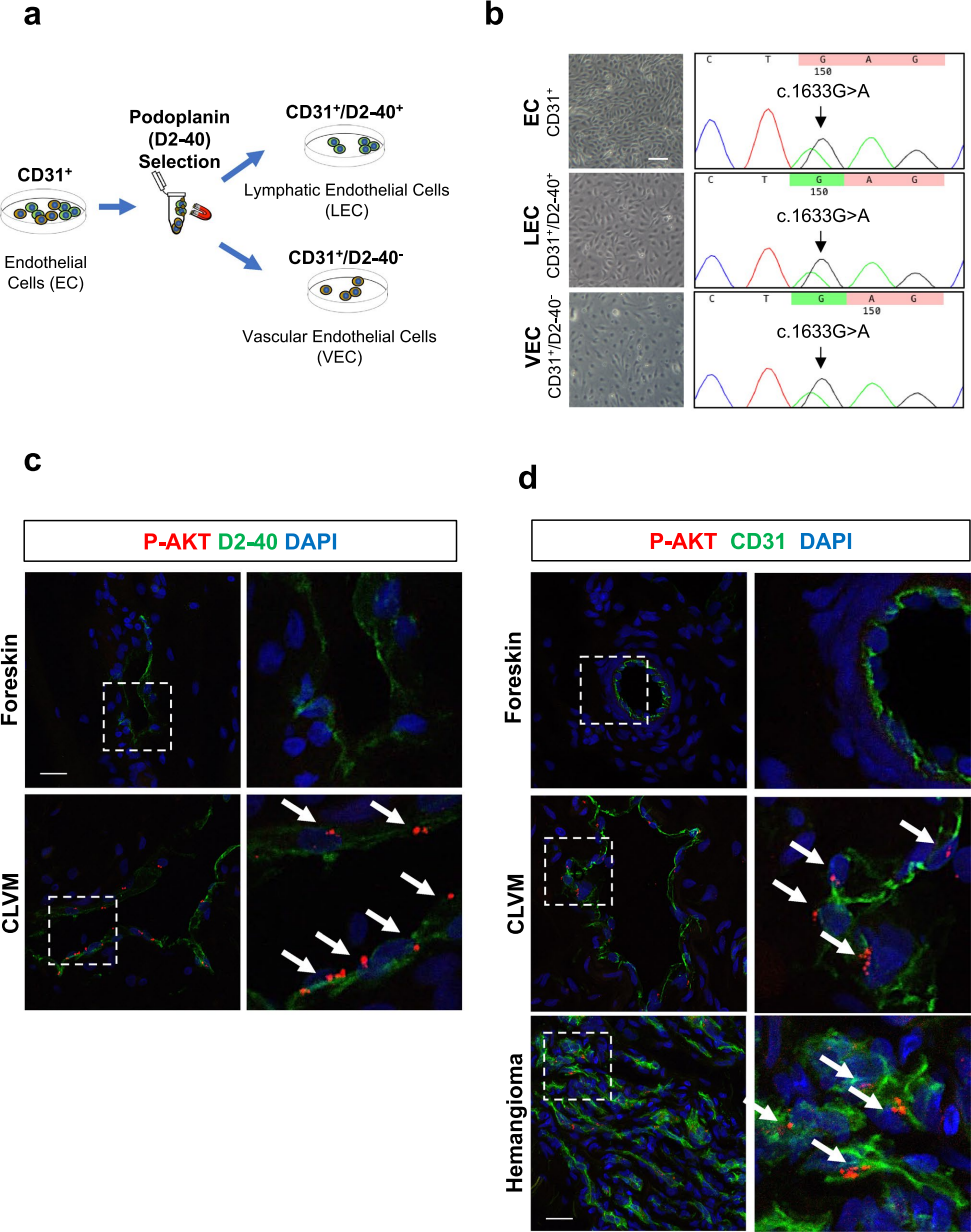
*PIK3CA* hotspot mutations identified in CLVM vascular and lymphatic EC. **a** Isolation strategy to separate CLVM EC (CD31^+^) into lymphatic EC (LEC) (CD31^+^/D2-40^+^) and vascular EC (VEC) (CD31^+^/D2-40^−^) populations using immunomagnetic bead sorting. **b** CLVM EC (CD31^+^), LEC (CD31^+^/D2-40^+^) and VEC (CD31^+^/D2-40^−^) from each patient were sequenced to compare the presence of somatic *PIK3CA* mutation. Representative images of CLVM A (c.1633G > A) EC, LEC and VEC cell morphology and sequencing chromatogram. Scale bar: 100 μm. **c** Tissue sections from patient CLVM and normal human foreskin stained with phospho-Ser473-AKT (red) and LEC marker D2-40 (green). **d** Tissue sections from patient CLVM, normal human foreskin and vascular tumor (hemangioma) stained with phospho-Ser473-AKT (red) and EC marker CD31 (green) (CD31^+^ blood vessel in CLVM image is negative for D2-40 as seen in adjacent section in Suppl. Fig. S6). Scale bars: 20 μm

To determine if the *PIK3CA* mutations were present in both LEC and VEC from CLVM lesions, EC were purified into CD31^+^/D2-40^+^ (LEC) and CD31^+^/D2-40^−^ (VEC) populations (Fig. 6a). Sanger sequencing showed that both LEC and VEC expressed the same heterozygous *PIK3CA* mutation identified in the corresponding patient’s EC (Fig. 6b and Table 2). This finding is supported by correspondingly increased phospho-AKT levels in the abnormal vasculature of CLVM patient tissue. Immunofluorescent staining revealed that both lymphatic (CD31^+^/D2-40^+^) and vascular (CD31^+^/D2-40^−^) channels expressed high levels of phospho-AKT (Fig. 6c and d and Suppl. Fig. S6), when compared to normal human foreskin tissue. Similar p-AKT staining pattern is also present in vascular tumor tissue (infantile hemangioma) (Fig. 6d).

**Table 2:** PIK3CA gene mutations

ID	EC (CD31^+^)	VEC (CD31^+^, D2-40^−^)	LEC (CD31^+^, D2-40^+^)	Peripheral Blood
A	E545K	E545K	E545K	–
B	H1047R	H1047R	H1047R	N/A
C	E545K	E545K	E545K	–
D	H1047R	H1047R	H1047R	–
E	E542K	E542K	E542K	–
F	E545A	E545A	N/A	N/A
G	H1047R	H1047R	H1047R	–

*EC* endothelial cell, *VEC* vascular EC, *LEC* lymphatic EC

“–” no PIK3CA mutation, N/A source not available

**Fig. 7:**
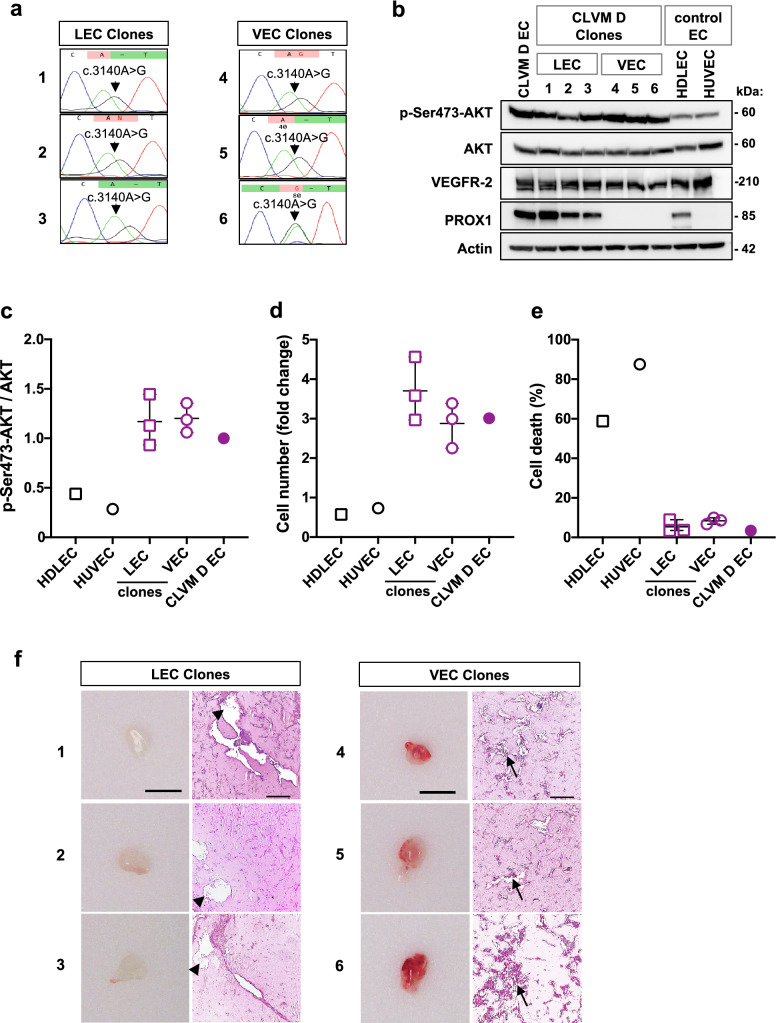
Clonal populations of CLVM LEC or VEC form vascular or lymphatic channels in mouse. **a** DNA Sanger sequencing chromatogram for 3 LEC and 3 VEC clones derived from CLVM D EC. **b** Immunoblot analysis of LEC and VEC clones for PI3K effector AKT (phospho-AKT (Ser473) and total AKT), pan-EC (VEGFR-2) and LEC-specific (PROX1) markers. **c** Densitometric quantification of phospho-AKT(Ser473) western blot bands relative to total AKT. Horizontal bar shows mean, data is normalized to CLVM D EC mean, *n* = 2 independent experiments. **d** Proliferation of LEC and VEC clones at 72 h in EBM-2 no FBS, normalized to time 0 (12 h after seeding). 3 LEC and 3 VEC clones were used. HUVEC and HDLEC served as normal controls. Horizontal bar shows mean (*n* = 5 technical repeats). **e** Resistance of CLVM D EC and clones to cell death induced by growth factor withdrawal at 72 h. HUVEC and HDLEC served as normal controls. Horizontal bar shows mean (*n* = 5 technical repeats). The cell death was measured as: [(number of cytotox+cells at 72 h)/ (total number of cells at 72 h) × 100]. **f** Representative images of 3 LEC and 3 VEC clones injected in vivo. Lesions were analyzed at 9 days for gross morphology and sections stained for H&E. *n* = 4 mice per each LEC or VEC clone. Arrowheads point to lymph fluid material inside the lymphatic channels and arrows point to red blood cells within the vascular channels. Scale bars: 1 cm (plug) and 100 μm (H&E)

Furthermore, we generated clonal populations of LEC and VEC from CLVM D EC. Each distinct clone (3 LEC clones and 3 VEC clones) showed expression of the *PIK3CA* p.H1047R mutation (Fig. 7a) and AKT hyper-phosphorylation (Fig. 7b, c). LEC and VEC clones also showed increased proliferation and resistance to cell death stimuli when compared to HDLEC and HUVEC (Fig. 7d, e).

When injected into the xenograft model, each LEC and VEC clonal population formed solely lymphatic or vascular lesions, respectively (Fig. 7f). While LEC clone-derived channels showed histological features of lymphatic fluid, VEC clone-derived vascular channels were filled with erythrocytes (Fig. 7f). All together, these data verify that somatic *PIK3CA* mutations affect both LEC and VEC in CLVM, and that in vivo each cell type gives rise to vasculature of the same identity (lymphatic or vascular).

## Discussion

In this study, we isolated EC populations from CLVM patients and identified cancer-associated hotspot *PIK3CA* mutations in EC from 7/7 patients. In vitro, CLVM EC showed increased AKT phosphorylation at Ser473 and Thr308, hyperproliferation, and pronounced resistance to cell death. This hyperproliferation was attenuated with both PIK3CA and AKT inhibitors. We established a novel murine xenograft model of CLVM by injecting patient-derived *PIK3CA*-mutant EC into immune-deficient mice. In the xenograft lesions, CLVM EC generated enlarged and abnormally shaped lymphatic and vascular channels, mimicking histological hallmarks of CLVM patient lesions. Furthermore, through both immunomagnetic isolation and single cell clonal expansion, we found that *PIK3CA* mutations were present in both LEC and VEC isolated from CLVM lesions.

CLVM are described as combined vascular anomalies by the classification criteria established by the International Society for the Study of Vascular Anomalies (ISSVA) [5]. Combined vascular anomalies involve abnormalities in more than one type of vascular channel. Somatic, mosaic *PIK3CA* mutations have been previously identified in CLVM patient tissue [17, 18, 23]. Interestingly, *PIK3CA* mutations have also been reported in simple vascular anomalies whose hallmarks are combined in CLVM, namely lymphatic malformation (LM) and venous malformation (VM) [18, 20, 21, 24, 25], whereas simple capillary malformations are most commonly associated with mutations in the GNAQ (G protein subunit alpha Q) gene [33–35]. Transgenic expression of the mutant PIK3CA p.H1047R in the developing mouse is embryonically lethal with severe vascular remodeling defects, even when the mutation is specifically targeted to EC via the Tie2 promoter [36]. These findings could explain the lack of inherited germline hotspot *PIK3CA* mutations in humans. In CLOVES tissue, the highest frequency of the *PIK3CA*-mutant allele is around 31% [17], strongly suggesting that not all of the cells within the CLVM lesion harbor the mutation. While *PIK3CA* mutations were shown to be expressed by EC (CD31^+^) in VM and by LEC (CD31^+^/D2-40^+^) in LM and generalized lymphatic anomaly (GLA) [20, 27, 29, 31], the cellular localization of the mutations in CLVM remains ill-defined. In a study by Kurek et al., most of the affected tissues in a CLOVES patient expressed the mutation, with high frequency in the abnormal vein and skin with lymphatics [17]. These data prompted our interest in characterizing the role of EC in CLVM pathogenesis.

In our study, EC from CLVM lesions were purified, characterized and examined for the presence of *PIK3CA* mutations. Our results demonstrate that activating *PIK3CA* variants were found in the EC in 7/7 CLVM patients. Additionally, EC populations from each CLVM patient were further separated into LEC and VEC by immunomagnetic separation (all patients) or by clonal expansion (CLVM D). Through both methodologies, we identified the same *PIK3CA* mutation and strong AKT activation in both LEC and VEC, strongly supporting the hypothesis that the mutation is acquired before lymphatic specification occurs during vascular development. Additionally, we showed that each LEC and VEC clonal populations generated lymphatic or vascular lesions, respectively. Of interest, precursors for LEC mainly reside in the lining of embryonic veins [1, 37], allowing us to speculate that the *PIK3CA* mutation first appears in a subset of VEC, some of which later differentiate into LEC. It is worth noting that the non-venous origin of LEC progenitors has also been documented in different mesenchymal compartments and presomitic mesoderm [38–40].

*PIK3CA* is one of the most common oncogenic mutations in cancer [26]. Interestingly, *PIK3CA* hotspot mutations are also found in a subset of overgrowth disorders which involve vascular malformations and are classified as PROS (PIK3CA-related overgrowth spectrum) [9, 22]. Despite the presence of the same somatic activating *PIK3CA* mutations identified in cancer, PROS and CLVM rarely acquire a malignant phenotype. An important difference between cancer and developmental disorders such as PROS, CLVM, VM and LM is that these congenital lesions are characterized by a single copy of the mutant *PIK3CA* (heterozygous), while human cancer often contains multiple oncogenic hits along the same pathway [41]. Heterozygous *PIK3CA* mutations most likely arise in progenitor cells during development. Conversely, cancer-causing mutations arise in differentiated cells, resulting in susceptibility to additional oncogenic mutations and a stem-like phenotype. To explain the broad spectrum of syndromes caused by heterozygous *PIK3CA* mutations, it is tempting to speculate that the mutational event occurs at different stages during embryonic development and therefore affects different types of cells [42]. Furthermore, it is still unclear if phenotype severity is dependent on the mutational activation level or amount of mosaicism.

PI3K activation is responsible for AKT phosphorylation via Phosphoinositide-dependent Kinase 1 (PDK1) [43]. AKT hyperactivation is a well-documented hallmark of patient-derived EC from LM and VM [20, 24, 27, 29]. Increased PI3K/AKT signaling is also a hallmark of pathological blood vessel growth and of tumor vasculature [44, 45]. Of interest, sustained AKT activation (myrAKT) in normal EC causes increased blood vessel size and recapitulates the structural abnormalities of tumor blood vessels [46]. Furthermore, several other mutations leading to increased PI3K/AKT pathway signaling (i.e., *TIE2, PTEN, AKT*) are also linked to vascular anomalies and overgrowth syndromes [47], providing evidence that AKT is a potential common therapeutic target for the treatment of these anomalies. In certain vascular malformations and tumors, the MAPK pathway is also affected as a consequence of genetic mutations (i.e., *GNAQ, RASA1, EPHB4, BRAF, NRAS, TIE2*) [47]. Several studies have shown that *PIK3CA*-mutated LEC isolated from LM display mildly increased MAPK activation [20, 27]. However, in our study, ERK1/2 phosphorylation levels in CLVM EC were comparable to normal EC. These results suggest that the MAPK pathway is unaffected in CLVM and likely not contributing to disease pathogenesis.

In vitro, we show that CLVM EC harboring *PIK3CA* mutations proliferated at a greater rate than normal LEC. It is particularly worth noting that CLVM EC consistently exhibited growth advantage and resistance to cell death in absence of growth factors and serum, while HDLEC and HUVEC cells rapidly died in these conditions. Since the discovery of *PIK3CA* mutations in cancer, pharmacologic inhibitors of PI3K, mTOR, and AKT are in different stages of clinical development and trial [48–50]. In particular, the PIK3CA inhibitor BYL719 and the AKT inhibitor ARQ092 are in clinical trials for patients with PROS and Proteus syndrome. These drugs demonstrate partial efficacy with reportedly tolerable side-effects [30, 51]. In our study, we took advantage of the hyperproliferative nature of CLVM EC to demonstrate that PI3K/AKT inhibitors suppress the mutant cell growth in a dose-dependent manner. However, in our experimental conditions, both BYL719 and ARQ092 also inhibit proliferation of control EC, suggesting patients using these drugs should be closely monitored for off-target toxicity, especially during events requiring physiological or reparative angiogenesis.

To date, the only published murine model of CLVM is based on the ubiquitous transgenic expression of a dominant active form of *PIK3CA* that was generated by fusion of the SH2 domain of the p85 subunit to p110 [30, 52, 53]. In our study, we utilized a clinically relevant approach based on patient-derived EC and resected tissue. We used patient-derived CD31^+^ EC populations, consisting of both *PIK3CA*-mutant LEC and VEC, to generate a xenograft model of CLVM that reflects the same *PIK3CA* genetic variants found in patients. These murine CLVM lesions exhibited aberrant, enlarged vascular and lymphatic channels, thereby mimicking key histological hallmarks of human CLVM tissue. It is important to note that, while each CLVM EC sample varies by the percentage of LEC versus VEC, this percentage/ratio does not necessarily reflect the composition of the entire patient lesion but rather the site-specific composition of the tissue specimen obtained.

## Electronic supplementary material

Below is the link to the electronic supplementary material.

Supplementary file1 (PDF 14200 kb)
