# Supplementary material for: Constitutively active PIK3CA mutations are expressed by lymphatic and vascular endothelial cells in capillary lymphatic venous malformation
Source: Angiogenesis. 2020 Apr 30;23(3):425–42. doi: 10.1007/s10456-020-09722-0 (PMC7311380; doi:10.1007/s10456-020-09722-0)
Supplement: Supplementary file 1 — Supplementary file1 (PDF 14200 kb) [file 10456_2020_9722_MOESM1_ESM.pdf]

Supplementary Data for

Constitutively active PIK3CA mutations are expressed by lymphatic and vascular endothelial cells in Capillary Lymphatic Venous Malformation

Timothy D. Le Cras[1, 2]*, Jillian Goines[3]#, Nora Lakes[3]#, Patricia Pastura[1], Adrienne M. Hammill[3, 4], Denise M. Adams[5], Elisa Boscolo[2, 3]*

[1]Division of Pulmonary Biology, [2]Department of Pediatrics, University of Cincinnati College of Medicine, [3]Division of Experimental Hematology and Cancer Biology, [4]Cancer and Blood Diseases Institute, Division of Hematology, Cincinnati Children's Hospital, Cincinnati, OH, USA
[5] Boston Children's HospitalDivision of Hematology/Oncology Harvard Medical School, Boston, MA, USA

*Corresponding author:
Elisa Boscolo, Division of Experimental Hematology, Phone:513-803-7267 Email: elisa.boscolo@cchmc.org
Tim Le Cras, Division of Pulmonary Biology, Phone:513-636-8151 Email: tim.lecras@cchmc.org
Cincinnati Children's Hospital Medical Center, 3333 Burnet Avenue, Cincinnati, Ohio 45229-3039

Supplemental Figures:

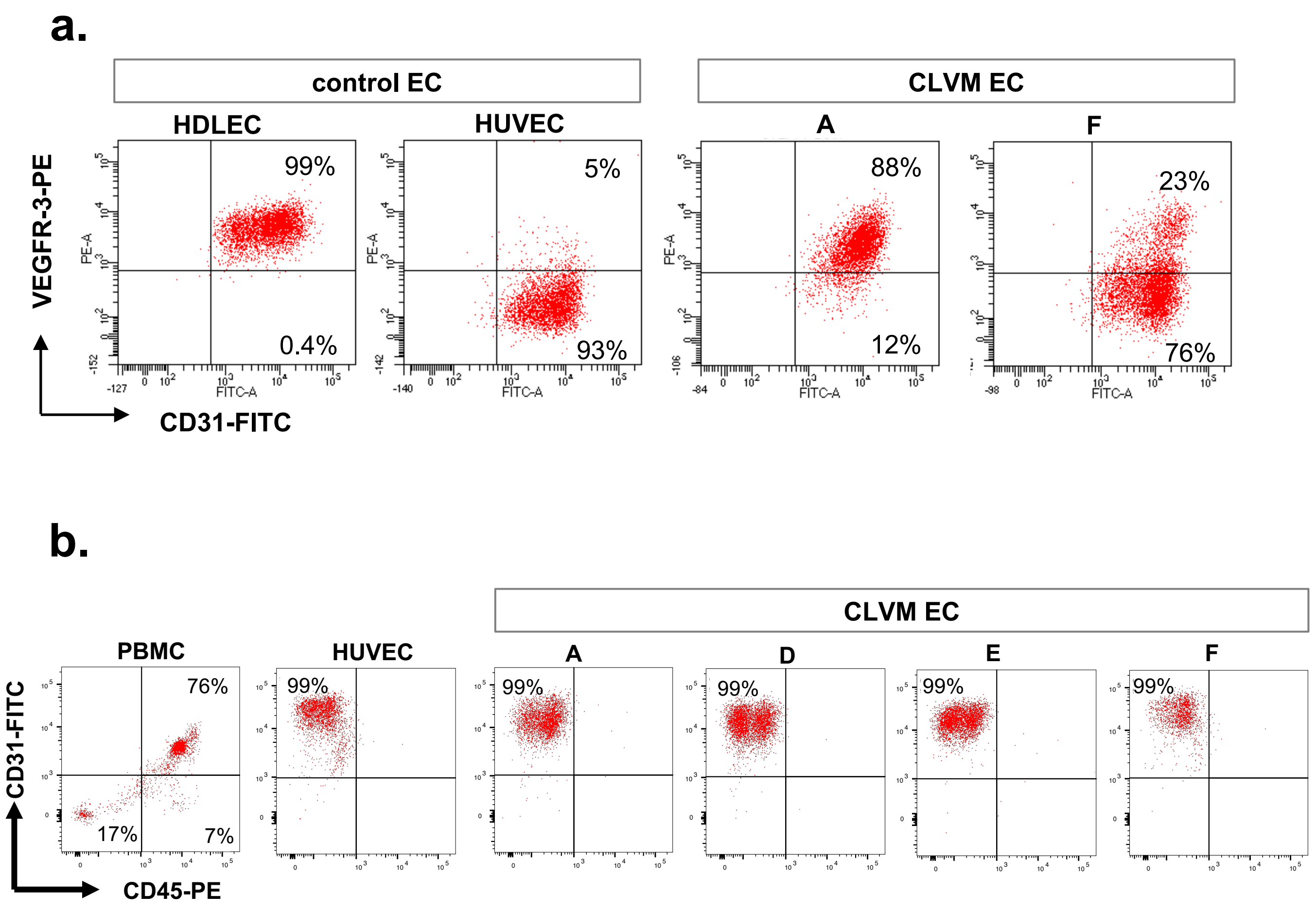

Supplemental Figure S1. a. Flow cytometric analysis of CLVM A and F EC, HUVEC, and HDLEC stained for EC marker CD31 and lymphatic EC marker VEGFR-3. **b.** Flow cytometric analysis of peripheral blood mononuclear cells (PBMC), HUVEC and CLVM EC stained for CD31 and hematopoietic cell marker CD45.

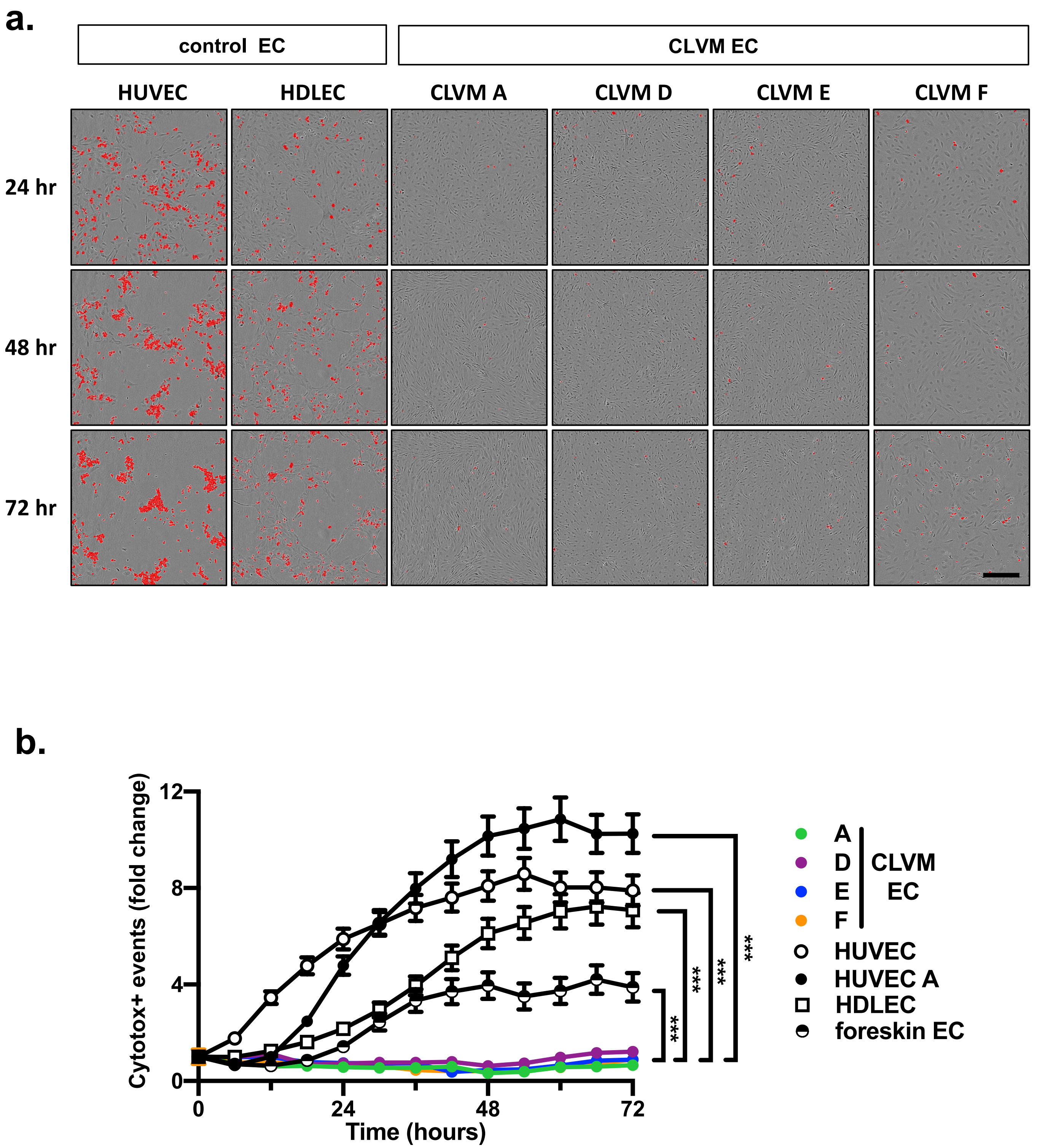

Supplemental Figure S2. CLVM EC are highly resistant to cell death induced by growth factor withdrawal. a. Contrast phase images of CLVM EC and control EC cultured for 96hr in basal medium without FBS. Dead cells are Cytotox^{+} (red). Scale bar: 300 μM. **b.** Resistance of CLVM EC and control EC to cell death induced by growth factor withdrawal (representative experiment, n=2). HUVEC: from a single donor, HUVECA: pooled from 5 different donors two-way ANOVA, ****P*<0.001

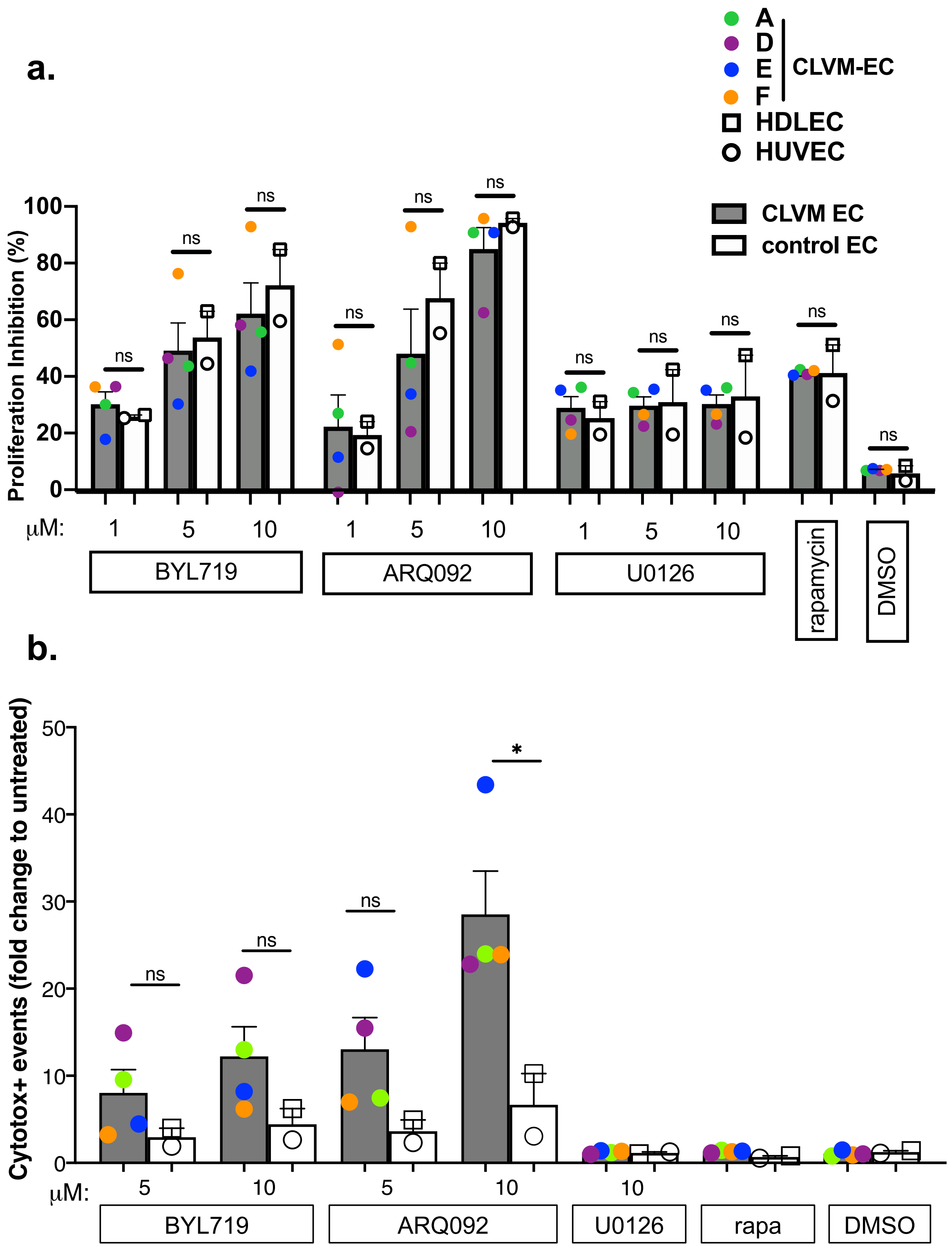

Supplemental Figure S3. PI3K-AKT inhibition in CLVM EC and control cells. a. CLVM EC (CLVM A, D, E, F) and control EC (HDLEC and HUVEC) treated for 48hs with PIK3CA inhibitor BYL719, AKT inhibitor ARQ092, MEK inhibitor U0126 (1,5,10μM) and rapamycin (15nM). Data is normalized to untreated control. The percent (%) inhibitory rate of compounds was calculated as [((OD_{540} untreated - OD_{540} compound) / (OD_{540} untreated)) * 100%]. **b.** Resistance to cell death of CLVM EC and control EC in response to drug treatment at 24hs. *P<0.05, ns: P>0.05.

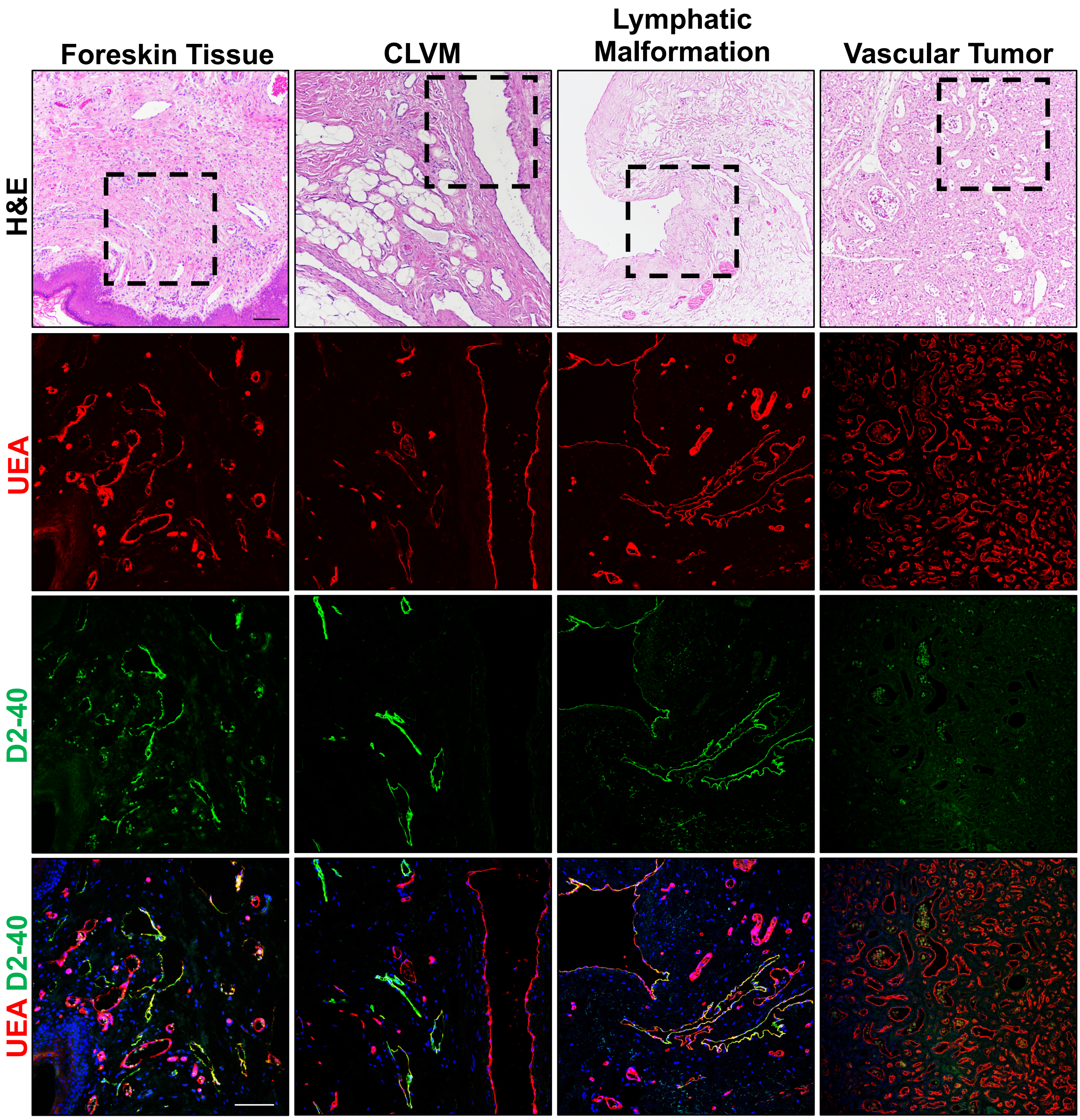

Supplemental Figure S4. Tissue explants from normal human foreskin, patient CLVM, lymphatic malformation and hemangioma (vascular tumor) were sectioned and stained with hematoxylin and eosin (H&E), EC marker UEA (red), and LEC marker D2-40 (green). Scale bars: 100μm.

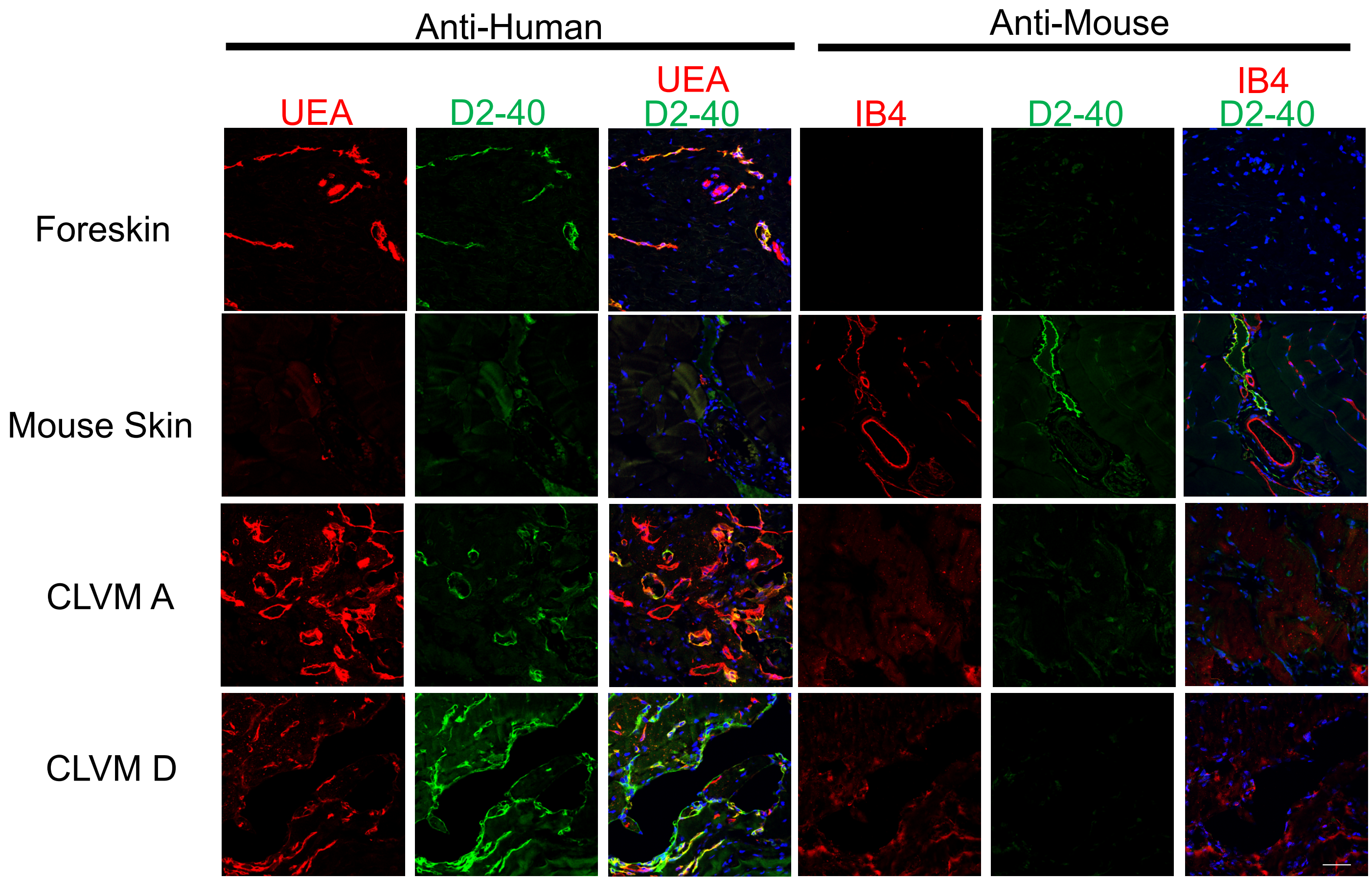

Supplemental Figure S5. Normal human foreskin, normal mouse skin, CLVM A lesion plug and CLVM D lesion plug were sectioned and consecutive sections stained with human EC marker UEA (red, left), human-specific LEC anti-D2-40 antibody (green, left), mouse EC marker IB4 (red, right), mouse-specific LEC anti-D2-40 antibody (green, right), and DAPI (blue). Scale bar: 50μm.

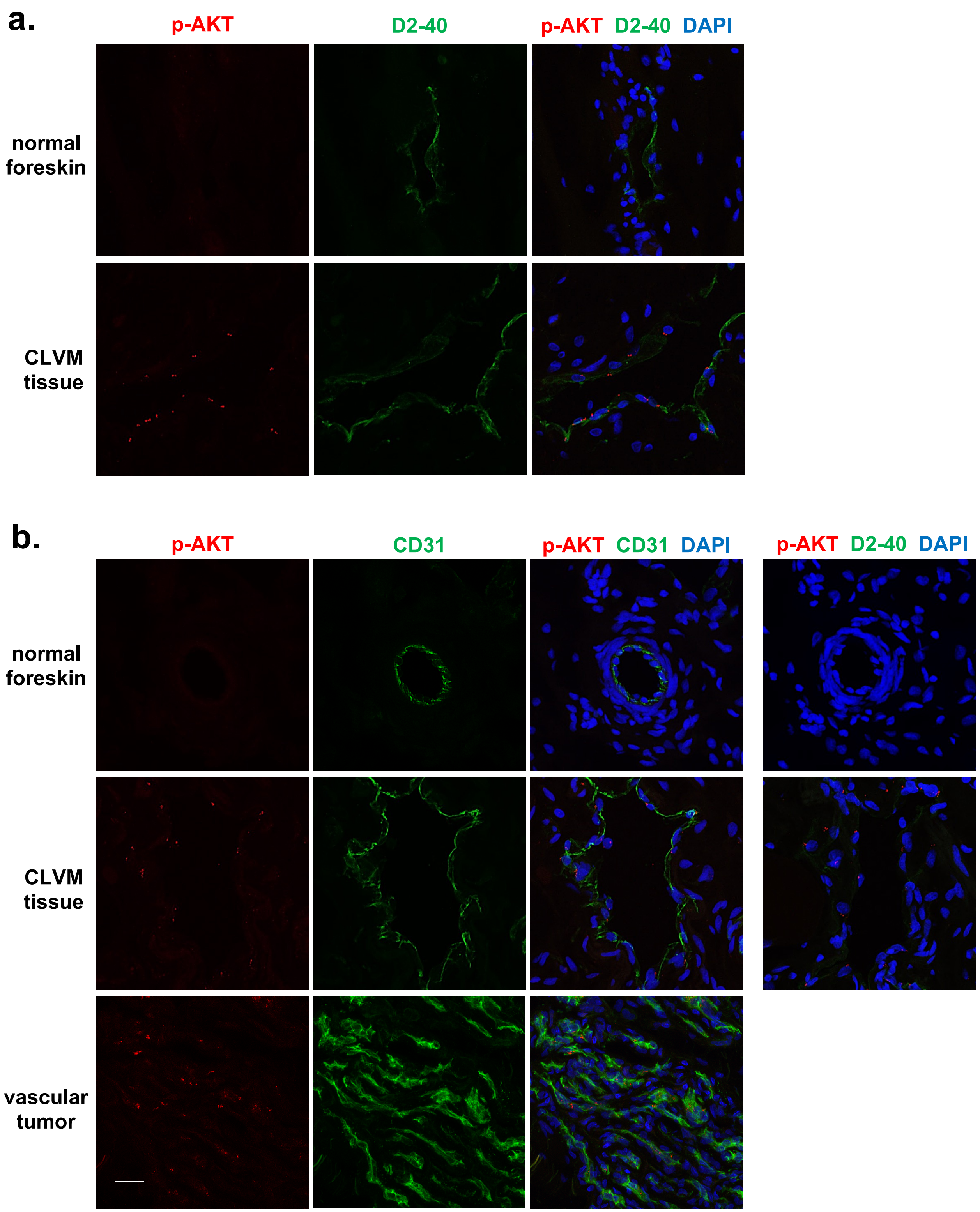

Supplemental Figure S6. Tissue sections from normal human foreskin, patient CLVM and vascular tumor (infantile hemangioma) stained with phospho-Ser473-AKT (red), LEC marker D2-40 (green) or EC marker CD31 (green). **a.** Single channel images relative to the overlay in Fig. 5c and **b.** in Fig 5d. On the right: consecutive section staining for D2-40 to show the channels imaged are vascular (VEC) and not lymphatic. Scale bar: 20μm.
